# Post Activation Potentiation Is Greater in Human Triceps Brachii Versus Triceps Surae Muscles

**DOI:** 10.1002/mus.70172

**Published:** 2026-02-06

**Authors:** William S. Zoughaib, Madison J. Fry, Ahaan Singhal, Richard L. Hoffman, Andrew R. Coggan

**Affiliations:** ^1^ Exercise Physiology Laboratory, Department of Kinesiology, School of Health & Human Sciences Indiana University Indianapolis Indianapolis Indiana USA

**Keywords:** muscle fiber type, myosin regulatory light chain, neuromuscular electrical stimulation, twitch contractile properties

## Abstract

**Introduction/Aims:**

Voluntary muscle contractions result in a temporary increase in twitch force, a phenomenon termed post activation potentiation (PAP). In rodents and other species, PAP is observed predominantly or exclusively in fast twitch muscles. However, it has been suggested that in humans, PAP occurs more or less independently of muscle fiber type.

**Methods:**

Eighteen healthy men and women (27 ± 8 years) underwent an electrical stimulation protocol during which two sets of four twitches were elicited both pre and post 6 s maximal voluntary contractions of the triceps surae (60%–70% slow twitch) and triceps brachii (60%–70% fast twitch) muscles.

**Results:**

Unpotentiated peak twitch torque (PTT) was higher in the larger triceps surae versus the smaller triceps brachii (i.e., 13.4 ± 5.3 vs. 3.4 ± 2.1 nm; *p* < 0.001), but time to peak torque was shorter (i.e., 84 ± 7 vs. 132 ± 14 ms; *p* < 0.001) and relative rate of torque development (RTD) was greater in the triceps brachii (2294 ± 257 vs. 1425% ± 102%/s; *p* < 0.001). PAP increased PTT by 172% ± 124% in the triceps brachii versus 20% ± 20% in the triceps surae (*p* < 0.001). Absolute RTD also increased more in the triceps brachii (i.e., 240% ± 170% vs. 31% ± 24%; *p* < 0.001). However, PAP‐induced changes in half‐relaxation time and relative rate of relaxation did not differ between muscle groups.

**Discussion:**

We conclude that PAP influences contraction but not relaxation of human muscle in a fiber type dependent manner. This should be kept in mind when interpreting individual differences in the results of neuromuscular testing, response to varying warm‐up protocols, etc.

**Abbreviations**
ANOVA: analysis of variance
ATP: adenosine triphosphate
HRT: half‐relaxation time
IPAQ: International Physical Activity Questionnaire
MET: metabolic equivalent of task
MVC: maximal voluntary contraction
PAP: post‐activation potentiation
P_i_
: inorganic phosphate
PTT: peak twitch torque
RLC: regulatory light chain
RR: rate of relaxation
RTD: rate of torque development
smMLCK: skeletal muscle myosin light chain kinase
TPT: time to peak torque

## 1 Introduction

Post‐activation potentiation (PAP) is the short‐term (i.e., seconds to minutes) enhancement of skeletal muscle function following prior voluntary contractile activity. Analogous to the post‐tetanic potentiation observed following electrically evoked contractions, PAP results in increases in the peak force and rate of force development during an isometric twitch [1]. Even greater increases in force (and hence power) are observed during concentric contractions [1]. These changes are thought to be largely, albeit not entirely, the result of a Ca^2+^‐stimulated increase in phosphorylation of the myosin regulatory light chain (RLC) [2]. This disrupts the superrelaxed state of myosin, leading to an increase in Ca^2+^ sensitivity and thus force upon stimulation [3]. Furthermore, although RLC phosphorylation has no apparent effect on the maximal ATPase activity or velocity of unloaded shortening of muscle [1], which are indicative of the rate of crossbridge cycling, the formation of more crossbridges results in an increase in the rate of force development when Ca^2+^ levels are subsaturating (e.g., during a twitch) [4].

In other species [5, 6, 7, 8, 9, 10], significant PAP is observed only in fast and not in slow muscles. At least in rodents, this appears to be primarily due to a > 10‐fold higher ratio of skeletal muscle myosin light chain kinase (smMLCK) activity to myosin light chain phosphatase activity in fast muscle fibers [11]. As a result, during contractions a marked increase in RLC phosphorylation occurs only in fast fibers, and in fact it has been suggested that the potentiated state is the normal operating state of fast muscles [12]. In humans, however, muscle contraction results in phosphorylation of the RLC in both fiber types [13, 14], and considerable PAP is observed even in muscles with a high percentage of slow fibers [15, 16, 17, 18, 19, 20]. Moreover, endurance athletes exhibit greater PAP in their trained muscles despite a slower phenotype [20]. Contrastingly, PAP has also been reported to be greater in faster vs. slower human muscles [19, 21] and to be inversely correlated with baseline (i.e., unpotentiated) twitch kinetics [21, 22], implying a fast twitch fiber dependence. Thus, the effects of muscle fiber type on PAP in humans are still uncertain [1].

In addition to altering the rate of force development, PAP may (or may not) alter the rate of relaxation. In rodent muscle, PAP slows the rate of relaxation from a steady force but enhances the rate of relaxation during a twitch [1], effects that appear to be independent of either RLC phosphorylation or muscle fiber type [22]. In humans, on the other hand, PAP has been reported to either reduce [15, 16, 18, 20], not change [16, 17, 22, 23], or even increase [24] twitch half‐relaxation time (HRT) in various muscle groups. The effects of PAP on relaxation of human muscle, especially with respect to fiber type, are therefore still unclear.

The purpose of the present study was to determine the effects of a conditioning activity designed to elicit PAP on the twitch contractile properties of two human muscles differing significantly in fiber type composition, that is, the triceps brachii (60%–70% fast twitch) and triceps surae (60%–70% slow twitch) muscles [25, 26]. We hypothesized that PAP would increase twitch force and kinetics in both muscle groups, but that these effects would be larger in the triceps brachii versus the triceps surae.

## 2 Methods

### 2.1 Participants

Healthy adults provided informed consent to participate in this study, which was approved by the Human Subjects Office at Indiana University. Both men and women were included because prior research has demonstrated that there is no sex‐related difference in twitch potentiation immediately after an isometric contraction [27, 28]. To avoid the potentially confounding influence of exercise training on PAP [20], highly active individuals (i.e., those performing > 3000 metabolic equivalent of task (MET)‐min/week of moderate physical activity or > 1500 MET‐min/week of vigorous physical activity based on the short‐form International Physical Activity Questionnaire (IPAQ; [29]) were excluded). Other exclusion criteria were age < 18 or > 44 years, current use of antibiotics, phosphodiesterase inhibitors, tobacco, or any supplements intended to increase muscle mass or function, diagnosis of epilepsy or presence of a pacemaker or other implantable cardiac device, resting blood pressure > 140/90 mmHg, or an answer of yes to any of the seven general health questions of the Physical Activity Readiness Questionnaire [30]. In addition, women were excluded if they had an irregular menstrual cycle (average length < 21 or > 35 days), had missed more than three consecutive periods in the last 12 months, or were pregnant, using hormonal contraceptives, or on hormone replacement therapy. We did not attempt to control for menstrual cycle phase since it has been reported to have no influence on twitch contractile properties [31, 32].

### 2.2 Experimental Procedures

A constant current stimulator (Digitimer DS 7AH, Hertfordshire, UK) was used to elicit unpotentiated and potentiated isometric twitch contractions of the triceps brachii and triceps surae, with the resultant torque measured at 1000 Hz using an isokinetic dynamometer (Biodex System 4 Pro, Biodex Medical Systems, Shirley, NY) and data acquisition system (Biopac MP160, Biopac, Goleta, CA). The order of testing of the two muscle groups was randomized between participants.

**FIGURE 1:**
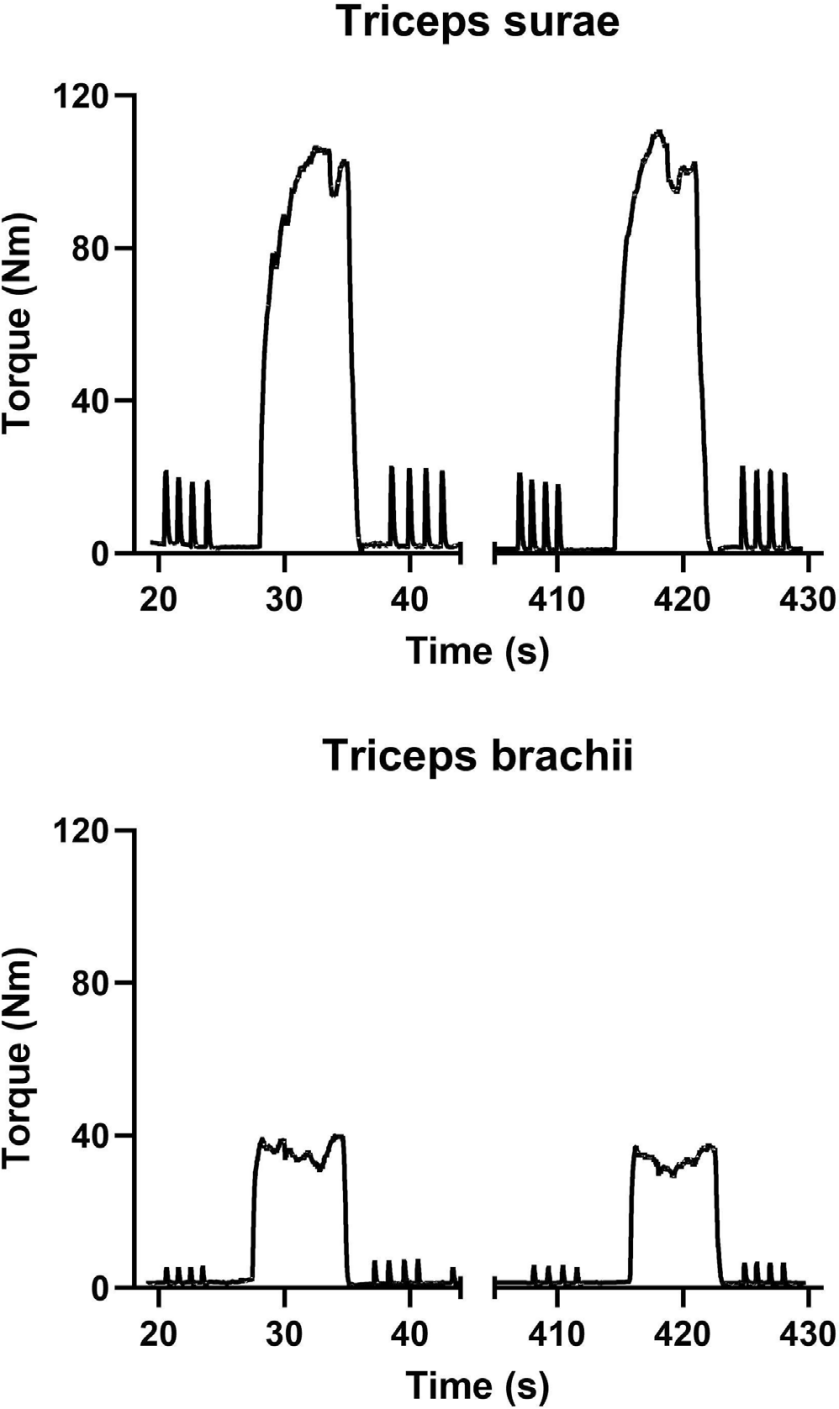
Effects of a 6 s maximal voluntary contraction (MVC) on twitch torque in the triceps surae (*top panel*) or triceps brachii (*bottom panel*) of a representative participant.

After shaving, abrading, and cleaning the participant's skin over the muscles of interest, two 5.08 × 8.89 cm self‐adhesive electrodes (Dura‐Stick Plus, Chattanooga Medical Supply, Chattanooga, TN) were applied to the first muscle to be tested. For the triceps brachii, the cathode was placed diagonally across the proximal posterolateral portion of the long and lateral heads, whereas the anode was placed transversely ~5 cm proximal to the olecranon process. For the triceps surae, the cathode was placed transversely over the gastrocnemius muscle ~5 cm distal to the popliteal fossa, whereas the anode was placed over the soleus muscle ~10 cm proximal to the calcaneus. The participant was then positioned on the dynamometer for testing of the triceps brachii (Figure S1, top panel) or triceps surae (Figure S1, bottom panel). Single stimuli (400 V, 200 μs) of increasing current were then applied at 3–5 s intervals until a plateau in twitch torque was observed. After a brief rest, four unpotentiated twitches were then elicited at approximately 1 s intervals. The participant then performed a 6 s maximal voluntary isometric contraction (MVC), during which strong verbal encouragement was provided. Previous research has demonstrated that MVCs of 3–10 s result in maximal PAP irrespective of muscle fiber type [16, 22]. Immediately afterwards, four potentiated twitches were elicited, also at approximately 1 s intervals. Results of a representative experiment are shown in Figure 1. After 10 min of rest [15, 23], a single stimulus was applied to verify reversal of the potentiation, then this sequence was repeated for that muscle. The electrodes were then moved, and the participant repositioned on the dynamometer to permit testing of the second muscle using the same procedures.

### 2.3 Data Analysis

Torque data were analyzed using Biopac AcqKnowledge version 5.08. The signal was first smoothed using a 10 ms rolling average filter, after which peak twitch torque (PTT), time to peak torque (TPT), and HRT were manually extracted for each twitch. Maximal rates of torque development (RTD) and relaxation (RR) were also determined based on the first derivative of the smoothed torque signal over a 20 ms interval. The eight values obtained for each of these parameters under each condition (i.e., unpotentiated and potentiated) were then averaged and these averages used in all subsequent calculations.

### 2.4 Statistical Analysis

Statistical analyses were performed using GraphPad Prism version 10.4.1 (build 627) (GraphPad Software, La Jolla, CA). Normality of data distribution was assessed using the D'Agostino and Pearson omnibus test. Two‐way (i.e., muscle × condition, both as within participant factors) analyses of variance (ANOVA) were used to compare twitch contractile properties between the triceps surae and triceps brachii. Differences between individual cell means were tested using the Holm‐Šidák multiple comparison procedure. The effects of PAP on percentage changes in PTT, RTD, and RR in the two muscle groups were compared using paired *t*‐tests. Pearson product correlations were used to evaluate the relationship between unpotentiated twitch characteristics and the extent of PAP. Multiplicity‐adjusted two‐tailed *P* values < 0.05 were considered statistically significant. Effect sizes are presented as Cohen's *d*.

## 3 Results

Eighteen individuals (15 men, three women) were studied. Their mean age, height, weight, and body mass index were 27 ± 8 years, 1.74 ± 0.07 m, 77.0 ± 18.3 kg, and 25.2 ± 4.8 kg/m^2^, respectively. Based on the IPAQ, they were moderately active, performing 1140 ± 751 MET‐min/week of physical activity.

**FIGURE 2:**
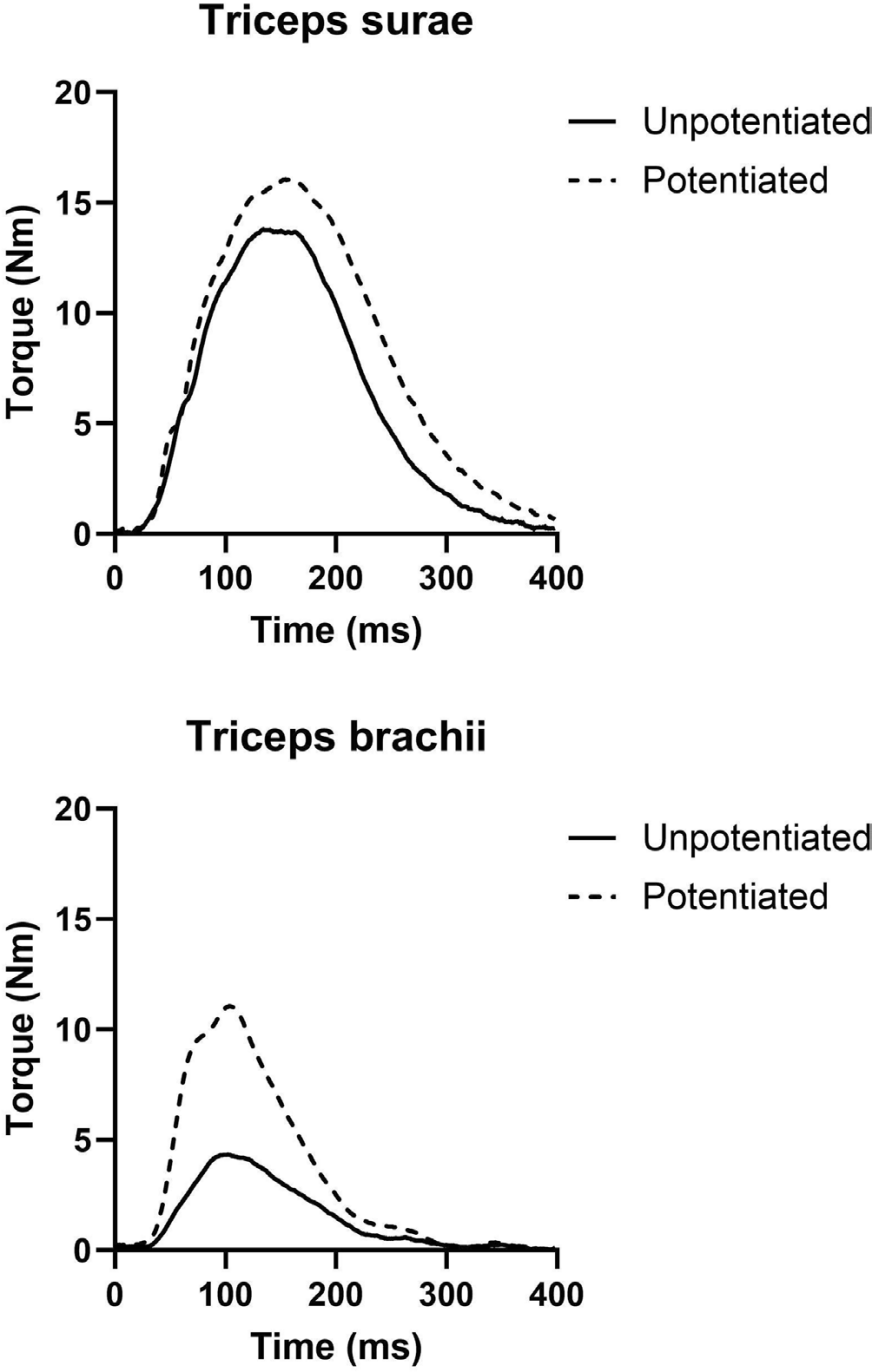
Effects of post‐activation potentiation on twitch torque in the triceps surae (*top panel*) and triceps brachii (*bottom panel*) of a representative participant.

The multiple (i.e., *n* = 8) twitches elicited under each condition (i.e., unpotentiated or potentiated) were used to calculate the 95% confidence limits for the mean values of PTT, TPT, HRT, absolute RTD, absolute RR, relative RTD, and relative RR. In relative terms these were determined to be ±3.2%, ±3.2%, ±4.5%, ±3.5%, ±4.1%, ±2.4%, and ±3.4% in the unpotentiated state and ±2.1%, ±2.7%, ±3.0%, ±2.5%, ±3.5%, ±1.8%, and ±2.9% in the potentiated state, respectively. Data from a representative participant are illustrated in Figure 2, whereas the contractile properties of the two muscle groups are summarized in Table 1.

**TABLE 1:** Twitch contractile properties of the triceps surae and triceps brachii muscle groups.

	Triceps surae			Triceps brachii		
	Unpotentiated	Potentiated	% Change	Unpotentiated	Potentiated	% Change
PTT (nm)	13.4 ± 5.3	15.8^b^ ± 5.9	+21 ± 20	3.4^c^ ± 2.1	7.6^b^, ^c^ ± 2.9	+172^d^ ± 124
TPT (ms)	132 ± 14	117^b^ ± 9	−11 ± 6	84^c^ ± 7	76^b^, ^c^ ± 9	−9 ± 10
Absolute RTD (nm/s)	191 ± 74	244^b^ ± 96	+31 ± 24	78^c^ ± 50	216^b^ ± 90	+240^d^ ± 170
Relative RTD (%/s)	1425 ± 102	1539^a^ ± 137	+8 ± 7	2294^c^ ± 257	2850^b^, ^c^ ± 350	+248^d^ ± 12
HRT (ms)	99 ± 13	96 ± 15	−2 ± 7	63^c^ ± 13	58^c^ ± 8	−3 ± 21
Absolute RR (nm/s)	−111 ± 45	−131^a^ ± 50	+21 ± 24	−41^c^ ± 28	−94^b^, ^c^ ± 40	+169^d^ ± 105
Relative RR (%/s)	−829 ± 82	−832 ± 105	0 ± 8	−1271^c^ ± 347	−1241^c^ ± 196	+3 ± 27

*Note:* Values are mean ± SD for *N* = 18. Potentiated versus unpotentiated for same muscle.

Abbreviations: HRT, half relaxation time; PTT, peak twitch torque; RR, rate of relaxation; RTD, rate of torque development; TPT, time to peak torque.

^a^ Potentiated vs. unpotentiated for same muscle: *p* < 0.05.

^b^ Potentiated vs. unpotentiated for same muscle: *p* < 0.001.

^c^ Triceps brachii vs. triceps surae for same condition: *p* < 0.001.

^d^ Percent change in triceps brachii vs. triceps surae: *p* < 0.001.

Based on ANOVA there was a significant interaction effect for PTT (i.e., F(1,17) = 6.779; *p* < 0.05), with post hoc testing indicating that the PTT of the larger triceps surae was significantly greater than that of the smaller triceps brachii in both the unpotentiated (i.e., *p* < 0.001, *d* = 4.891) and the potentiated (i.e., *p* < 0.001, *d* = 4.022) state. However, PAP increased PTT more in the triceps brachii than in the triceps surae in both absolute and relative terms (i.e., *p* < 0.05, *d* = 0.6135 and *p* < 0.001, *d* = 1.163).

There was also a significant interaction effect for TPT (i.e., F(1,17) = 6.419; *p* < 0.05), with TPT being significantly shorter in the triceps brachii both before (i.e., *p* < 0.001, *d* = 5.683) and after (i.e., *p* < 0.001, *d* = 4.839) potentiation. Potentiation also shortened TPT in both muscle groups, with the absolute decrease being greater in the triceps surae (i.e., *p* < 0.05, *d* = 0.6106). However, the relative change did not differ between them (i.e., *p* = 0.4800, *d* = 0.1700).

Significant interaction effects were also observed for absolute and relative RTD (i.e., F(1,17) = 25.10; *p* < 0.001 and F(1,17) = 36.69; *p* < 0.001). Based on post hoc testing, absolute RTD was significantly higher in the triceps surae during unpotentiated (i.e., *p* < 0.001, *d* = 2.235) but not during potentiated (i.e., *p* = 0.0559, *d* = 0.5648) twitches. On the other hand, relative RTD was higher in the triceps brachii both in the absence (i.e., *p* < 0.001; *d* = 3.973) and the presence (i.e., *p* < 0.001, *d* = 5.991) of PAP. The absolute RTD was accelerated to a greater degree by potentiation in the triceps brachii versus the triceps surae, both in absolute and relative terms (i.e., *p* < 0.001, *d* = 1.181 and *p* < 0.001, *d* = 1.159, respectively). The relative RTD also increased more in the triceps brachii, both absolutely and as a percentage (i.e., *p* < 0.001, *d* = 1.428 and *p* < 0.001, *d* = 1.068, respectively).

Main effects for muscle (i.e., F(1,17) = 94.68; *p* < 0.001) and condition (i.e., F(1,17) = 5.108; *p* < 0.05) were found for HRT, but the interaction effect was not significant. Absolute and the relative changes in HRT due to PAP also did not differ between the two muscle groups (i.e., *p* = 0.6156, *d* = 0.1205 and *p* = 0.8450, *d* = 0.0004, respectively).

There was a significant interaction effect for absolute RR (i.e., F(1,17) = 21.75; *p* < 0.001), with post hoc testing revealing that absolute RR was significantly more rapid in the triceps surae both when unpotentiated (i.e., *p* < 0.001, *d* = 3.320) and when potentiated (i.e., *p* < 0.001, *d* = 1.765). However, potentiation enhanced the absolute RR more in the triceps brachii than in the triceps surae, both in absolute and relative terms (*p* < 0.001, *d* = 1.099 and *p* < 0.001, *d* = 1.404).

Finally, relative RR showed a main effect for muscle (i.e., F(1,17) = 50.93; *p* < 0.001), but the condition and interaction effects were not significant. PAP had no impact on the relative RR in either muscle, with both the absolute and relative changes being non‐significant (i.e., *p* = 0.7081, *d* = 0.3049 and *p* = 0.6986, *d* = 0.1039).

**FIGURE 3:**
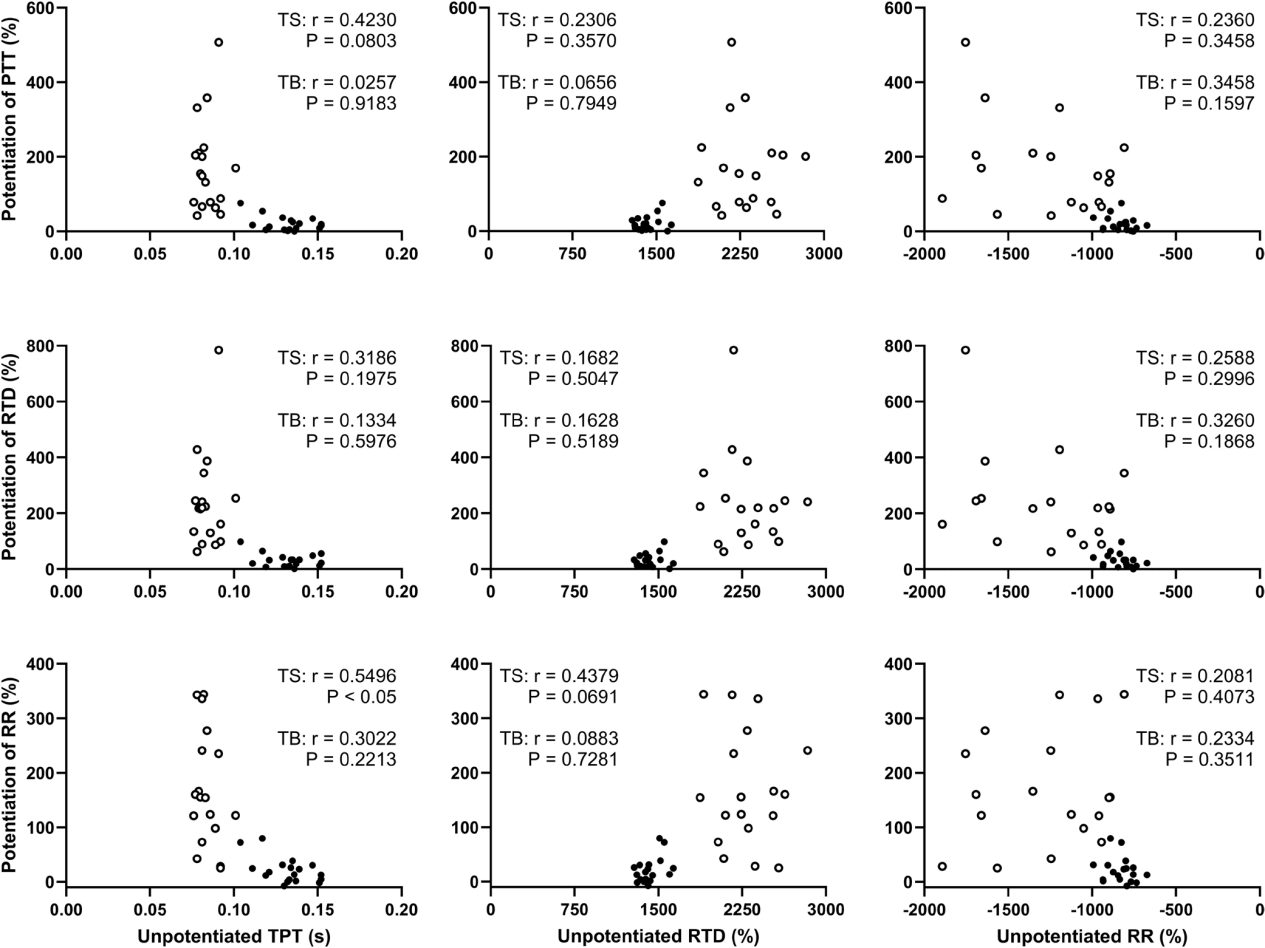
Relationship between potentiated‐induced changes in peak twitch torque (PTT, top row), rate of torque development (RTD, middle row), and rate of relaxation (RR, bottom row) with the unpotentiated time to peak torque (TPT, left column), RTD, and RR in individual participants. closed circles, triceps surae (TS). open circles, triceps brachii (TB).

As described above, the effects of potentiation on PTT and absolute RTD and RR were, on average, 8‐fold larger in the triceps brachii versus the triceps surae. However, the magnitudes of these changes were not related to baseline, that is, unpotentiated, twitch kinetics on an individual basis (Figure 3). The only exception was the potentiation‐induced increase in the rate of relaxation of the triceps surae, which was modestly correlated the unpotentiated TPT of this muscle.

## 4 Discussion

In the present study we found that performance of a 6 s MVC increased PTT and enhanced the absolute and relative RTD in both the predominantly fast twitch triceps brachii and the predominantly slow twitch triceps surae, but that these effects were significantly larger in the triceps brachii. Prior contraction also shortened TPT and HRT and increased the absolute but not the relative RR in both muscle groups. These observations support and expand current understanding of the fiber type‐specific effects of PAP on the twitch contractile properties of human muscle.

At least two previous studies have observed greater increases in PTT following a brief MVC in faster versus slower muscles of humans [19, 21]. However, the effects of PAP on PTT have also been reported to be similar in the triceps brachii and the triceps surae [20], or even larger in the tibialis anterior (75% slow twitch; [27]) versus the triceps surae [16]. Although the reason for these discrepant findings is not clear, our results are comparable to the former studies [19, 21], with the relative increase in PTT due to a 6 s MVC being > 8‐fold larger in the triceps brachii versus the triceps surae. Thus, in humans as in other species, PAP seems to increase twitch force to a much greater extent in fast muscle. Similarly, the greater increases in absolute and relative RTD that we observed in the triceps brachii versus the triceps surae are consistent with the results of animal studies indicating that PAP also increases the rate of force development primarily or exclusively in fast fibers [1]. In contrast, previous studies of the effects of PAP in fast versus slow human muscles have either not measured twitch kinetics [19, 21] or have only measured TPT and have found similar relative changes in both fast and slow muscles [16, 20]. PAP has a smaller effect on TPT than on RTD ([23]; Table 1), however, which may explain the latter findings.

In addition to the above‐described changes in PTT, TPT, and RTD, we also found that PAP was accompanied by a shorter HRT and a faster absolute RR, with the latter effect being greater in the triceps brachii than in the triceps surae. In relative terms, however, there were no changes in RR in either muscle group. The latter results imply that PAP does not alter the fundamental determinant of relaxation, i.e., the rate of dissociation of myosin from actin [33], in either fast or slow human muscle, with the higher absolute RR simply reflecting the detachment of more such crossbridges per unit time. This conclusion is consistent with the fact that RLC phosphorylation does not alter the maximal rate of crossbridge cycling, as indicated by ATPase activity or unloaded shortening velocity [1]. On the other hand, the shorter HRT in the potentiated state may be because twitch relaxation is a complicated process influenced by factors in addition to the rate of crossbridge detachment [33]. For example, it has been suggested that PAP may accelerate relaxation via increases in inorganic phosphate (*P*_i_) during the conditioning contraction, thus favoring “backward” transition of still‐attached crossbridges from their force‐generating to their non‐force‐generating state [33]. Although *P*_i_ would be expected to have increased less in the triceps surae versus the triceps brachii [34], reversal of the myosin power stroke is exquisitely sensitive to *P*_i_, even in cardiac muscle [35]. Increases in *P*_i_ during the MVC therefore could have accelerated and shortened the initial, linear phase of relaxation in the triceps surae as well as the triceps brachii. Alternatively, the greater PTT in the potentiated state may lead to more rapid “give” of the longest sarcomere in a myofibril and hence more rapid onset of the fast, that is, “chaotic”, phase of relaxation in both muscle groups [33, 36].

The above results therefore support the conclusion that PAP influences the mechanisms of force development but not relaxation in human muscle in a fiber‐type specific manner. Notably, however, with the exception of a weak correlation between unpotentiated TPT and the relative increase in RR in the triceps surae, no significant relationships were found between baseline twitch contractile properties and the extent of PAP on an individual basis. These results contrast with previous studies that have reported modest‐but‐significant correlation between the increase in PTT due to PAP and, for example, TPT in the unpotentiated state [21, 22, 24]. Although the reason(s) for the lack of any such relationships in the present study cannot be determined, the absence of a strong correlation (including in previous studies [21, 22, 24]) indicates that factors other than fiber type (e.g., differences in tendon stiffness [15]) must also contribute to intraindividual differences in the effects of PAP.

As with all studies, there are limitations to the present investigation. The most obvious is the fact that muscle biopsies were not obtained to verify the fiber type distribution of the triceps surae and triceps brachii in the present participants. However, differences in fiber type between these muscles are well‐established [25, 26]. This conclusion is reinforced by the differing contractile properties (e.g., TPT) that we observed. Nonetheless, this may have contributed to the apparent lack of any relationship between individual muscle characteristics and the degree of PAP. Furthermore, despite clearly differing in fiber type, both the triceps surae and triceps brachii are still mixed muscles, such that it is not possible to definitively ascribe our observations to strictly just slow twitch or fast twitch muscle fibers. We also did not quantify PAP in other muscles, at different joint angles/muscle lengths, in different states of fatigue, at different time points, and/or during dynamic muscle contractions, all of which can influence the magnitude of the response [17, 21, 22, 37, 38]. Likewise, we did not assess the effects of different conditioning contraction regimens, for example, the duration and/or number of MVC, which can differentially effect the degree of PAP in faster and slower muscles due to the greater fatigability of the former [16, 23].

In summary, in the present study we found that the effects of PAP on PTT and absolute and relative RTD, but not on HRT or relative RR, are greater in the predominantly fast‐twitch triceps brachii than in the predominantly slow‐twitch triceps surae of humans. We therefore conclude that, as in other species, PAP influences muscle contraction but not relaxation in humans in a fiber type dependent manner.

## Author Contributions

R.L.H. and A.R.C. conceived and designed research. W.S.Z. and A.R.C. analyzed data. W.S.Z, M.J.F., A.S., R.L.H., and A.R.C. performed experiments. W.S.Z. and A.R.C. interpreted results of experiments. W.S.Z. and A.R.C. prepared figures. W.S.Z. drafted manuscript. A.R.C. edited and revised the manuscript. W.S.Z, M.J.F., A.S, R.L.H., and A.R.C. approved final version of the manuscript.

## Funding

The authors have nothing to report.

## Ethics Statement

We confirm that we have read the Journal's position on issues involved in ethical publication and affirm that this report is consistent with those guidelines.

## Conflicts of Interest

The authors declare no conflicts of interest.

## Supporting information


**Figure S1:** Participant positioning when testing the triceps brachii (*top panel*) and triceps surae (*bottom panel*). For the triceps brachii, participants were positioned on the dynamometer with a chair back angle of 1.48 rad (85°) and with the shoulder of the dominant arm adducted and at an elevation of 0 rad (0°). The elbow was supported at a joint angle of 1.57 rad (90°) and straps were placed over the participant's waist, torso, and forearm to restrict extraneous movement. A plastic and fabric brace (Roylan Ulnar Deviation Splint, Performance Health, Cedarburg, WI) and an elastic wrap were used to prevent any movement of the wrist or fingers relative to the handgrip. For the triceps surae, the participant was positioned on the dynamometer with a chair back angle of 0.96 rad (55°) and the knee and ankle joints of the ipsilateral leg of the dominant arm at an angle of 0 rad (0°). Straps were placed over the waist and thigh as well as across the instep and metatarsals to prevent any movement of the leg or the foot with respect to the footplate.

## Data Availability

The data that support the findings of this study are available on request from the corresponding author. The data are not publicly available due to privacy or ethical restrictions.
