# Supplementary material for: Post Activation Potentiation Is Greater in Human Triceps Brachii Versus Triceps Surae Muscles
Source: Muscle Nerve. 2026 Feb 6;73(5):815–21. doi: 10.1002/mus.70172 (PMC13047951; doi:10.1002/mus.70172)
Supplement: Supplementary file 1 — Figure S1: Participant positioning when testing the triceps brachii (top panel) and triceps surae (bottom panel). For the triceps brachii, participants were positioned on the dynamometer with a chair back angle of 1.48 rad (85°) and with the shoulder of the dominant arm adducted and at an elevation of 0 rad (0°). The elbow was supported at a joint angle of 1.57 rad (90°) and straps were placed over the participant's waist, torso, and forearm to restrict extraneous movement. A plastic and fabric brace (Roylan Ulnar Deviation Splint, Performance Health, Cedarburg, WI) and an elastic wrap were used to prevent any movement of the wrist or fingers relative to the handgrip. For the triceps surae, the participant was positioned on the dynamometer with a chair back angle of 0.96 rad (55°) and the knee and ankle joints of the ipsilateral leg of the dominant arm at an angle of 0 rad (0°). Straps were placed over the waist and thigh as well as across the instep and metatarsals to prevent any movement of the leg or the foot with respect to the footplate. [file MUS-73-815-s001.docx]

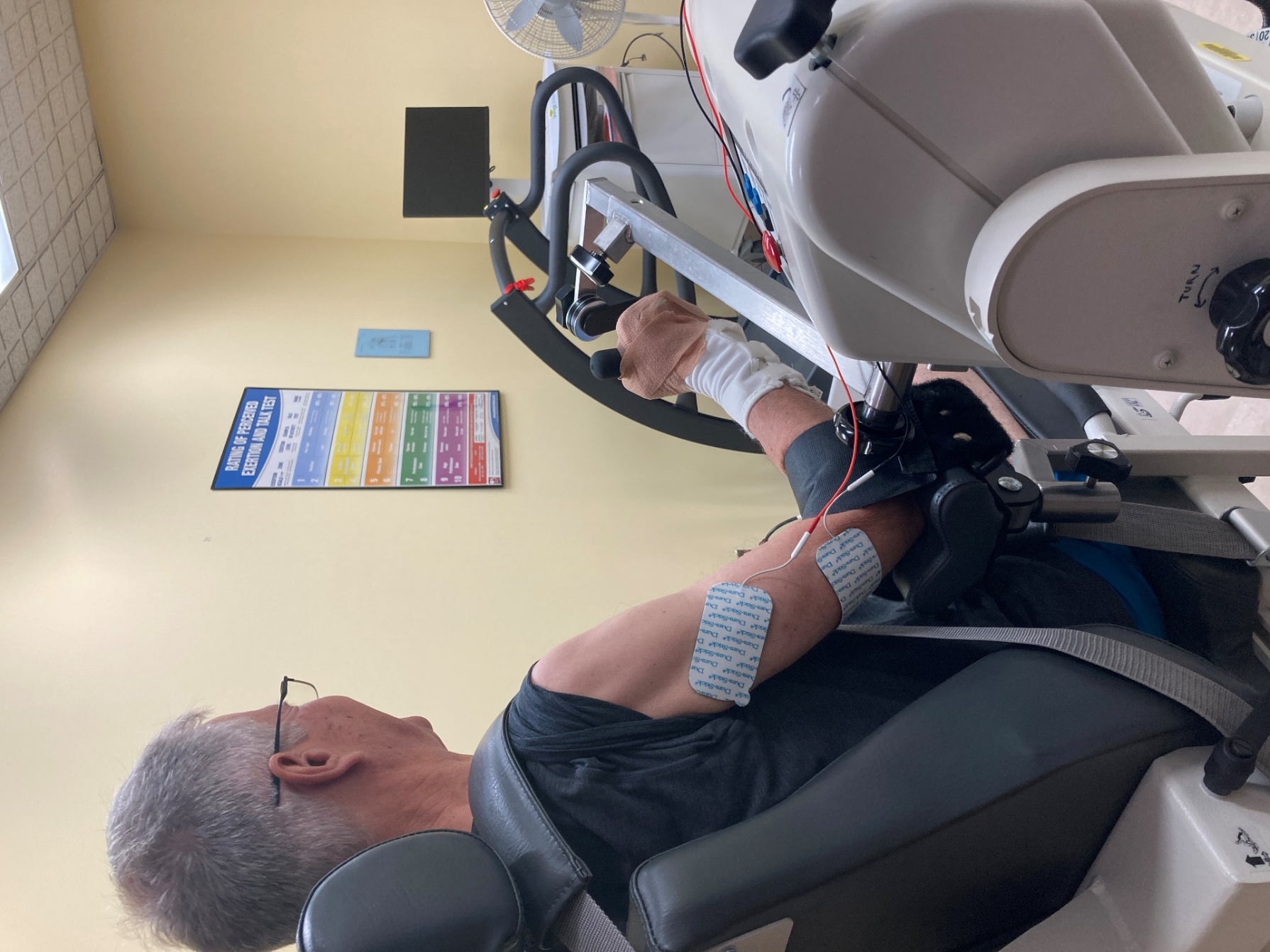


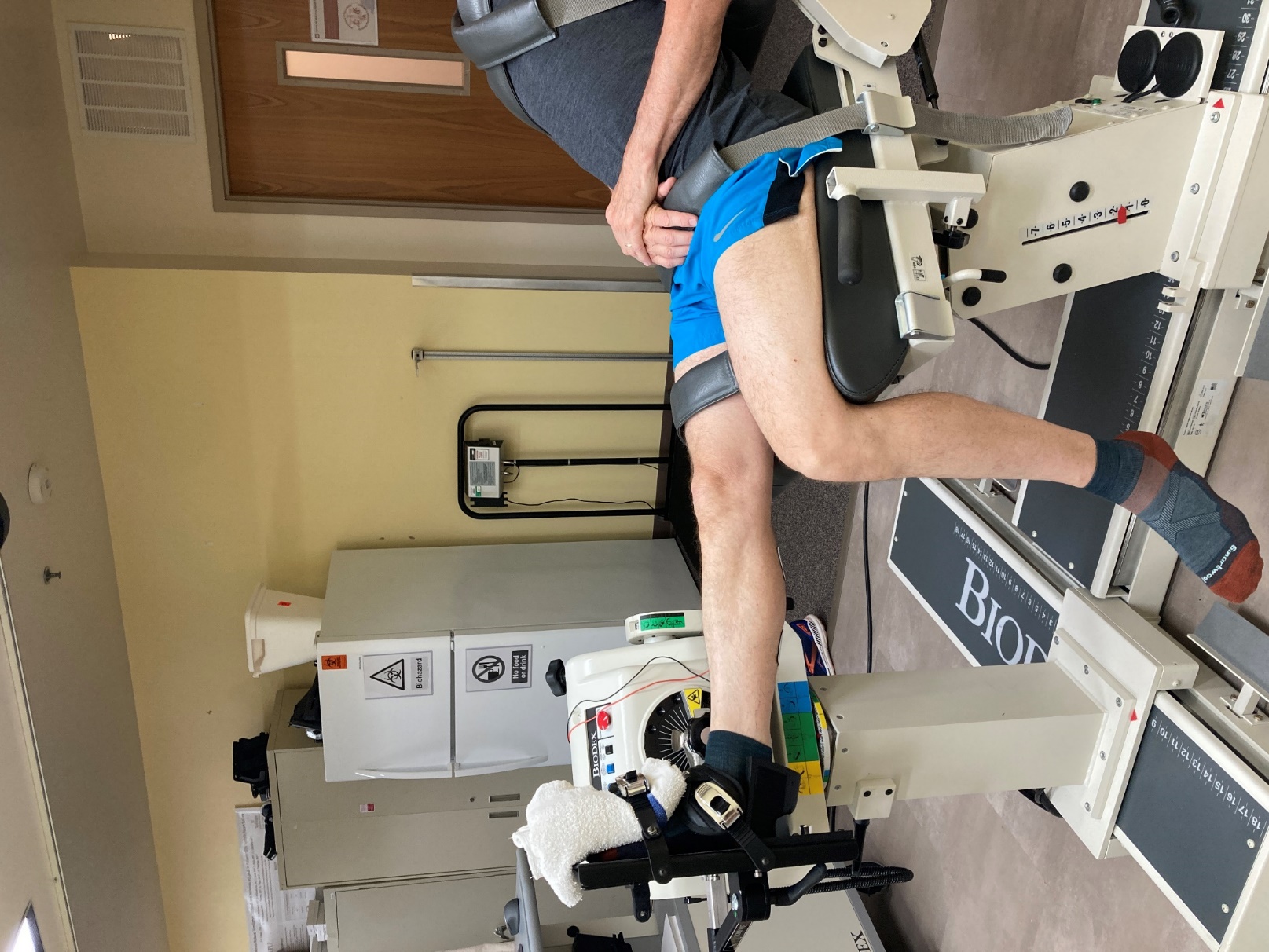


Supplemental Figure 1. Participant positioning when testing the triceps brachii (*top panel*) and triceps surae (*bottom panel*). For the triceps brachii, participants were positioned on the dynamometer with a chair back angle of 1.48 rad (85°) and with the shoulder of the dominant arm adducted and at an elevation of 0 rad (0°). The elbow was supported at a joint angle of 1.57 rad (90°) and straps were placed over the participant’s waist, torso, and forearm to restrict extraneous movement. A plastic and fabric brace (Roylan Ulnar Deviation Splint, Performance Health, Cedarburg, WI) and an elastic wrap were used to prevent any movement of the wrist or fingers relative to the handgrip. For the triceps surae, the participant was then positioned on the dynamometer with a chair back angle of 0.96 rad (55°) and the knee and ankle joints of the ipsilateral leg of the dominant arm at an angle of 0 rad (0°). Straps were placed over the waist and thigh as well as across the instep and metatarsals to prevent any movement of the leg or the foot with respect to the footplate.
